# Pre-snaring endoscopic mucosal resection: a simplified alternative to precutting technique for large sessile serrated lesions

**DOI:** 10.1055/a-2871-7987

**Published:** 2026-05-21

**Authors:** Pan Xu, LiYun Fu, Wan Zhou

**Affiliations:** 1 Department of Clinical Laboratory, Medical Sciences Research Center 568864 University-Town Hospital of Chongqing Medical UniversityChongqingChina; 2 Endoscopy Center 568864 University-Town Hospital of Chongqing Medical UniversityChongqingChina; 3 Department of Gastroenterology 568864 University-Town Hospital of Chongqing Medical UniversityChongqingChina

A sessile serrated lesion (SSL) in the cecum can be difficult to resect when it is large and flat 1 , as conventional endoscopic mucosal resection (EMR) may be limited by inadequate snare positioning due to the flat morphology and indistinct margins 2 . Precutting EMR has been proposed to facilitate snare placement by performing a circumferential mucosal incision. However, this technique can be technically demanding, particularly for less experienced endoscopists, and carries a risk of perforation during mucosal incision 3 . Herein, we present a simplified technique termed pre-snaring EMR ( Video 1 ).

Pre-snaring EMR for a large sessile serrated lesion, including cold snare precutting, submucosal injection, en bloc resection, and clip closure. EMR, endoscopic mucosal resection.Video 1

**Fig. 1:**
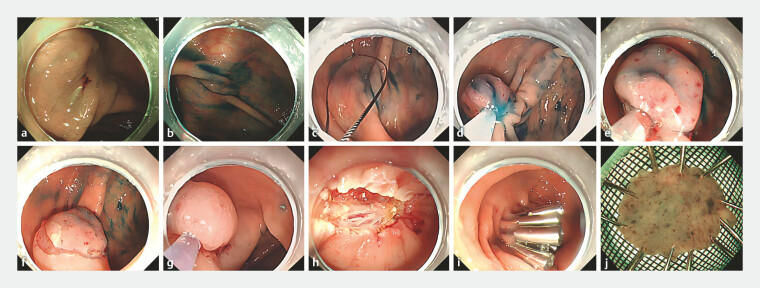
Stepwise demonstration of the pre-snaring EMR technique. **a** and **b** A 1.8-cm SSL in the cecum, clearly delineated after indigo carmine spraying. ( **c** and **d** Cold snaring of the lesion margin. **e** and **f** Submucosal injection with adequate lifting; visible exposure of the submucosal layer after cold snaring. **g** Snare resection along the preformed groove using electrocautery. **h** Complete resection without bleeding or perforation. **i** Closure of the mucosal defect with clips. **j** The resected specimen is stretched and pinned for the assessment of completeness. EMR, endoscopic mucosal resection.

A 59-year-old woman was found to have a SSL measuring approximately 18 mm in the cecum. After delineation of the lesion margins using indigo carmine, a snare was positioned around the lesion and gradually closed without electrocautery to achieve the cold transection of the surrounding mucosa, thereby exposing the submucosal layer and creating a groove for subsequent snare placement. Submucosal injection was then performed, followed by standard EMR along the preformed groove. En bloc resection was achieved without immediate or delayed complications ( Fig. 1 ).

Compared with conventional precutting EMR, this technique may simplify the procedure and lower the technical threshold, while potentially reducing the risks associated with electrosurgical incision; however, its safety and efficacy still require further validation.

Endoscopy_UCTN_Code_TTT_1AQ_2AD
